# A Rational Engineering Strategy for Designing Protein A-Binding Camelid Single-Domain Antibodies

**DOI:** 10.1371/journal.pone.0163113

**Published:** 2016-09-15

**Authors:** Kevin A. Henry, Traian Sulea, Henk van Faassen, Greg Hussack, Enrico O. Purisima, C. Roger MacKenzie, Mehdi Arbabi-Ghahroudi

**Affiliations:** 1 Human Health Therapeutics Portfolio, National Research Council Canada, 100 Sussex Drive, Ottawa, Ontario, Canada, K1A 0R6; 2 Human Health Therapeutics Portfolio, National Research Council Canada, 6100 Royalmount Avenue, Montreal, Quebec, Canada, H4P 2R2; 3 School of Environmental Sciences, University of Guelph, 50 Stone Road East, Guelph, Ontario, Canada, N1G 2W1; 4 Department of Biology, Carleton University, 1125 Colonel By Drive, Ottawa, Ontario, Canada, K1S 5B6; US Naval Research Laboratory, UNITED STATES

## Abstract

Staphylococcal protein A (**SpA**) and streptococcal protein G (**SpG**) affinity chromatography are the gold standards for purifying monoclonal antibodies (**mAbs**) in therapeutic applications. However, camelid V_H_H single-domain Abs (**sdAbs** or **V**_**H**_**Hs**) are not bound by SpG and only sporadically bound by SpA. Currently, V_H_Hs require affinity tag-based purification, which limits their therapeutic potential and adds considerable complexity and cost to their production. Here we describe a simple and rapid mutagenesis-based approach designed to confer SpA binding upon *a priori* non-SpA-binding V_H_Hs. We show that SpA binding of V_H_Hs is determined primarily by the same set of residues as in human mAbs, albeit with an unexpected degree of tolerance to substitutions at certain core and non-core positions and some limited dependence on at least one residue outside the SpA interface, and that SpA binding could be successfully introduced into five V_H_Hs against three different targets with no adverse effects on expression yield or antigen binding. Next-generation sequencing of llama, alpaca and dromedary V_H_H repertoires suggested that species differences in SpA binding may result from frequency variation in specific deleterious polymorphisms, especially Ile57. Thus, the SpA binding phenotype of camelid V_H_Hs can be easily modulated to take advantage of tag-less purification techniques, although the frequency with which this is required may depend on the source species.

## Introduction

Therapeutic antibodies (**Abs**) represent the fastest-growing class of biologic drugs, with expanding applications in cancer, chronic diseases and autoimmunity (reviewed in [1–3]). Currently licensed biologics are most commonly fully human or humanized monoclonal Abs (**mAbs**), with antigen-binding fragments such as Fab, F(ab’)_2_ and scFv making up a smaller proportion of the market [4]. Next-generation Ab therapeutics will likely exploit the improved functional properties of molecularly engineered Abs, including bispecific Abs, Ab-drug conjugates and Fc-variant Abs [1,4,5]. V_H_H single-domain antibodies (**V**_**H**_**Hs**), the variable domains of heavy-chain-only Abs produced naturally by camelid ungulates, may be useful in the design of next-generation biologics as a result of their small size, stability and modularity [6].

Full-length mAbs of all IgG subtypes can be easily purified by affinity chromatography, taking advantage of the high-affinity interactions between the IgG Fc and either staphyloccal protein A (**SpA**) or streptococcal protein G (**SpG**) [7]. Purification of Ab fragments bearing light chains can be accomplished using protein L resins but purification of V_H_Hs can be more complex, often requiring recombinant fusion to an affinity tag sequence [8]. The presence of affinity tags may impact protein folding, stability, solubility and aggregation state either directly or indirectly *via* the elution process [9–14]; although such tags can be removed proteolytically after purification, this process adds time and cost to the production pipeline. The gold standard for tag-less purification of Ab fragments and V_H_Hs is affinity chromatography using staphyloccal protein A (**SpA**; reviewed in [15]), which contains binding sites for both the IgG Fc region and variable regions of Ig heavy chains, with the specificity of the latter being restricted to human IGHV3 gene family products [16–20] and their homologues in experimental animal species [21,22]. The structural basis for binding of IGHV3 Abs by SpA was initially ascribed broadly to residues in FR3, FR1 and possibly CDR2 [23,24] and later defined more precisely using a co-crystal structure of a human IGHV3-encoded IgM in complex with SpA [25]. Binding appears to depend on thirteen Ig variable region residues at the direct interface with SpA, which are almost universally conserved in germline IGHV3 genes, but can be lost as a result of somatic hypermutation [17–19]. The importance of these residues has been inferred from structural studies and comparison with sequences of non-SpA-binding Abs, but never formally tested by mutagenesis.

Both Old World and New World camelids produce both conventional and heavy-chain-only Abs, which are rearranged during B-cell development from separate sets of germline variable genes (V_H_ and V_H_H genes, respectively [26]). Most camelid V_H_H genes are homologous to human IGHV3-family genes and fall into 4–7 subfamilies [26,27], although recent work suggests that human IGHV4 homologues may also exist that can be rearranged as both conventional and heavy-chain only Abs [28]. Despite their homology to human IGHV3-family genes, it has been reported that the majority of camelid V_H_Hs are not bound by SpA [29,30], although this may depend to some degree on species origin. At the amino acid level, the major sequence differences between camelid V_H_Hs and human IGHV3-family variable domains are (*i*) the presence of hallmark V_H_H solubilizing residues located in FR2 at the interface with the absent V_L_ domain [27], (*ii*) an elevated frequency of intradomain disulfide bridges between CDR3 and other regions, which may constrain V_H_H structure [31], (*iii*) the presence of various other germline polymorphisms, depending on the individual V_H_H gene and (*iv*) high somatic mutation rates [26]. However, it is unclear whether and how any of these sequence characteristics should impact SpA binding. Thus, the purpose of this work was: (*i*) to identify V_H_H residues required for SpA binding and evaluate their overlap with those defined for human IGHV3 Abs [25], and (*ii*) to develop a strategy to confer SpA binding upon *a priori* non-binding V_H_Hs without compromising antigen binding. Our data confirm that SpA binding by V_H_Hs is determined primarily by the same set of contact positions as in human IGHV3 mAbs, but also provide a comprehensive description of IGHV domain polymorphisms that are permissive and non-permissive for SpA binding. These data can be used to engineer SpA-binding V_H_Hs using limited site-specific mutagenesis, with no adverse effects on expression yield or antigen binding.

## Materials and Methods

### Phage-displayed libraries and isolation of V_H_Hs

The V_H_Hs described in this report are directed against a variety of antigens and were initially isolated from phage-displayed V_H_H libraries for reasons other than the study of SpA binding, as described previously [32–38].

### V_H_H mutagenesis

The amino acid sequences of five non-SpA-binding camelid V_H_Hs (ICAM11-4, ICAM34-1 [39], IGF1R-4, IGF1R-5 [40] and AFAI [35]) were altered at specific FR positions, either individually or in combination, either for humanization purposes or to confer SpA binding. Constructs encoding the engineered V_H_Hs were synthesized commercially in the pSJF2H expression vector ([31]; GenScript, Piscataway, NJ or Life Technologies, Carlsbad, CA).

### Soluble V_H_H monomer and pentamer expression and purification

Wild-type or engineered V_H_H genes bearing *BbsI/BamHI* or *BbsI*/*ApaI* restriction sites were cloned into pSJF2H or pVT2 expression vectors (for monomeric and pentameric expression, respectively) as described [32,33,35]. 6×His- and c-Myc-tagged V_H_H monomers and pentamers were expressed in *E*. *coli* TG1, extracted from the periplasm by osmotic shock and purified by immobilized metal affinity chromatography (**IMAC**) or protein A chromatography using using HisTrap HP or HiTrap Protein A HP columns, respectively (GE Healthcare, Piscataway, NJ; [32,33]). The integrity and aggregation status of soluble V_H_H monomers and pentamers were assessed by SDS-PAGE, Western blotting and size exclusion chromatography [32,33].

### Surface plasmon resonance (SPR)

For screening of V_H_H monomers and pentamers for binding to immobilized SpA at a single concentration, analyte proteins were used directly after purification by immobilized metal affinity chromatography. For determination of binding affinities to immobilized antigen or SpA, V_H_H monomers were purified by size exclusion chromatography using Superdex^™^ 75 or 200 10/300 GL columns (GE Healthcare) on an ÄKTA FPLC protein purification system (GE Healthcare), and the monomer peaks collected in HBS-EP buffer (10 mM 4-(2-hydroxyethyl)-1-piperazineethanesulfonic acid (**HEPES**), pH 7.4, 150 mM NaCl, 3mM ethylenediaminetetraacetic acid (**EDTA**), 0.005% (v/v) surfactant P20; [32,33]). Briefly, either SpA (Thermo Fisher, Waltham, MA), human ICAM-1 ectodomain (R&D Systems, Minneapolis, MN) or human IGF1R ectodomain (R&D Systems) were immobilized on CM5 sensor chips using an amine coupling kit (GE Healthcare) in 10 mM acetate buffer, pH 4.5, with surface densities of 609, 1348 and 476 resonance units, respectively. V_H_H monomers and pentamers were injected at 25°C in HBS-EP buffer at a flow rate of 20 μL/min on a Biacore 3000 instrument (GE Healthcare) at different concentration ranges, depending on the interaction (binding to SpA: 50 nM—25 μM; binding to ICAM-1: 0.5 nM– 200 nM; binding to IGF1R: 0.1 nM– 10 nM). All surfaces were regenerated using 10 mM glycine, pH 2.0. Data were analyzed using BIAevaluation 4.1 software (GE Healthcare) and for affinity determinations, fitted to a 1:1 binding model.

### *In silico* scanning mutagenesis of the SpA:V_H_H interaction

The co-crystal structure of a human IGHV3-encoded Fab in complex with domain D of SpA (PDB ID: 1DEE) was used as a starting point for virtual scanning mutagenesis. Only one copy of the IGHV3 domain (chain D) and the bound SpA fragment (chain G, corresponding to SpA domain D positions Asp8-Lys58 as numbered by Graille *et al*. [25]) were retained. The IGHV3 domain was first “camelized” by introducing four mutations in FR2: Val37Phe, Gly44Glu, Leu45Arg and Trp47Ala. Hydrogen atoms were added to the resulting V_H_H:SpA complex and adjusted to maximize H-bonding interactions. Structural refinement of the complex was then carried out by energy-minimization using the AMBER force-field [41,42] with a distance-dependent dielectric and infinite cutoff for non-bonded interactions. Non-hydrogen atoms were restrained at their crystallographic positions with harmonic force constants of 20 and 5 kcal/(mol^.^A^2^) for the backbone and side-chain atoms, respectively. The resulting structure was then used for single-point scanning mutagenesis simulations at the following positions of the IGHV3 domain: 15, 17, 19, 57, 59, 64, 65, 66, 68, 70, 75, 81, 82a, and 82b (all positions using Kabat numbering). We used three protocols (SIE-SCWRL [43–45], FoldX [46,47] and Rosetta [48,49]) for modeling the structures and evaluating the energies of single-point substitutions of the other 17 naturally-occurring amino acids (Cys and Pro excluded) at each of these 14 positions relative to the wild-type sequence. A consensus approach over specific versions of these three protocols was applied for building and scoring IGHV3 mutants. Further technical and implementation details of this approach and its component methods can be found in Sulea *et al*. [50].

### Next-generation DNA sequencing

Phagemid replicative form DNA from naïve and immune phage-displayed V_H_H libraries was isolated from *E*. *coli* TG1 cells using QIAprep spin miniprep kits (QIAGEN, Valencia, CA). Next-generation sequencing libraries were generated by two-step PCR amplification of V_H_H genes and purified as previously described [51,52]. The final amplicons were pooled and purified from 1% (w/v) agarose gels using a QIAquick^®^ gel extraction kit (QIAGEN), desalted using Agencourt AMPure XP beads (Beckman-Coulter, Pasadena, CA), then sequenced on a MiSeq Sequencing System (Illumina, San Diego, CA) using a 500-cycle MiSeq Reagent Kit V2 and a 5% PhiX genomic DNA spike. From each sample, 0.1–2.1 million reads were generated, of which 0.4×10^5^–6.0×10^5^ were used for analysis after assembly using FLASH (default parameters; [53]) and quality filtering using the FAST-X toolkit with a stringency of Q30 over ≥95% of each read [54].

## Results

### Definition of V_H_H residues involved in SpA binding

**Fig 1:**
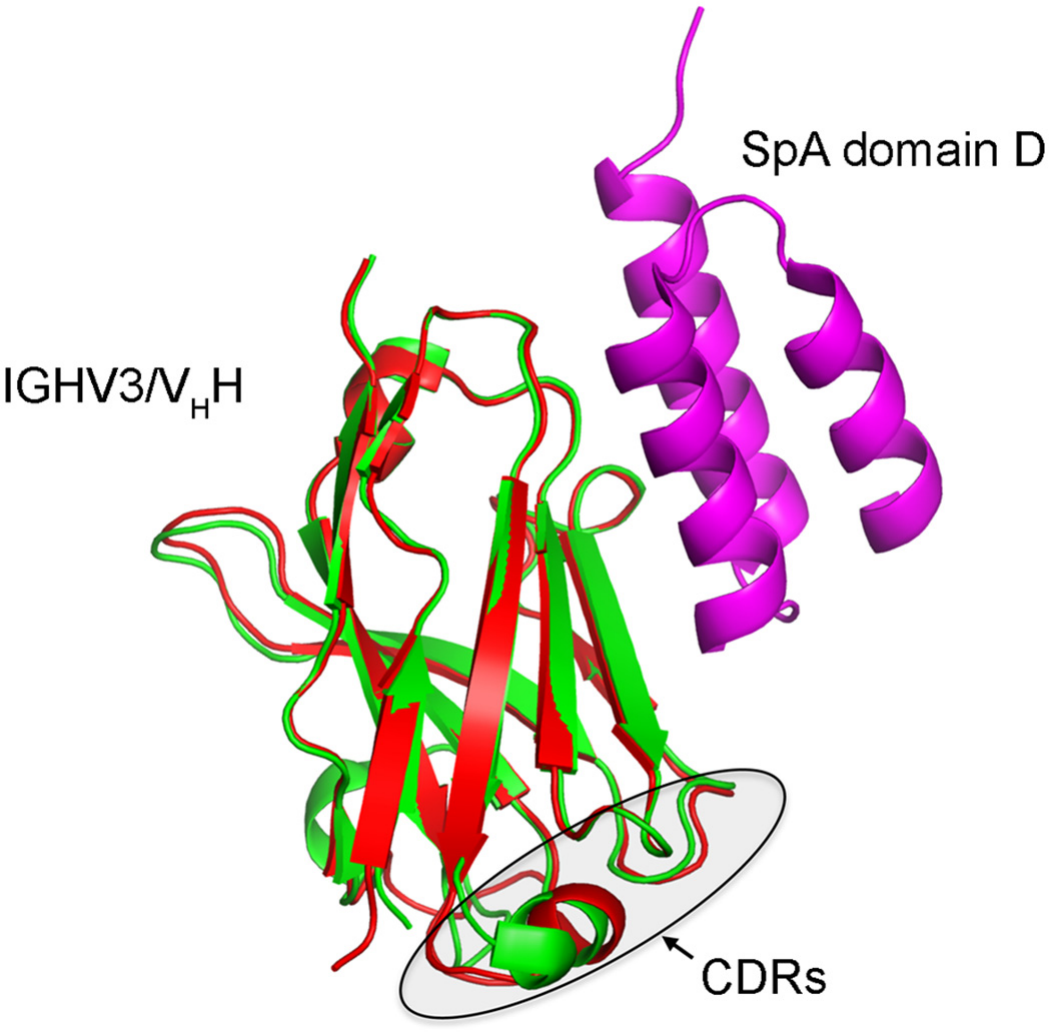
Overlay of three-dimensional structures of a llama V_H_H (green, PDB ID: 4NC0) and a human IGHV3 heavy chain variable domain (red, PDB ID: 1DEE) in complex with SpA domain D. Antigen-binding CDRs are indicated.

Previous structural work [25] suggested the existence of a set of seven core SpA binding residues in human IGHV3 heavy-chain variable regions (positions 19, 65, 66, 68, 70, 81 and 82a; all positions using Kabat numbering), with a lesser contribution of six additional contact residues (positions 15, 17, 57, 59, 64 and 82b). Parenthetically, these tend to be mostly conserved in SpA-binding human and murine Abs and altered *via* germline variation or somatic hypermutation in non-SpA-binding Abs [17–19], although neither the importance of each residue nor the spectrum of tolerated substitutions has been rigorously tested by mutagenesis studies. Overlay of the three-dimensional structures of a human IGHV3-encoded IgM heavy-chain variable domain [25] and a llama V_H_H directed against *Clostridium difficile* toxin A [55] showed strong overall conservation of these immunoglobulin folds (Fig 1).

**Fig 2:**
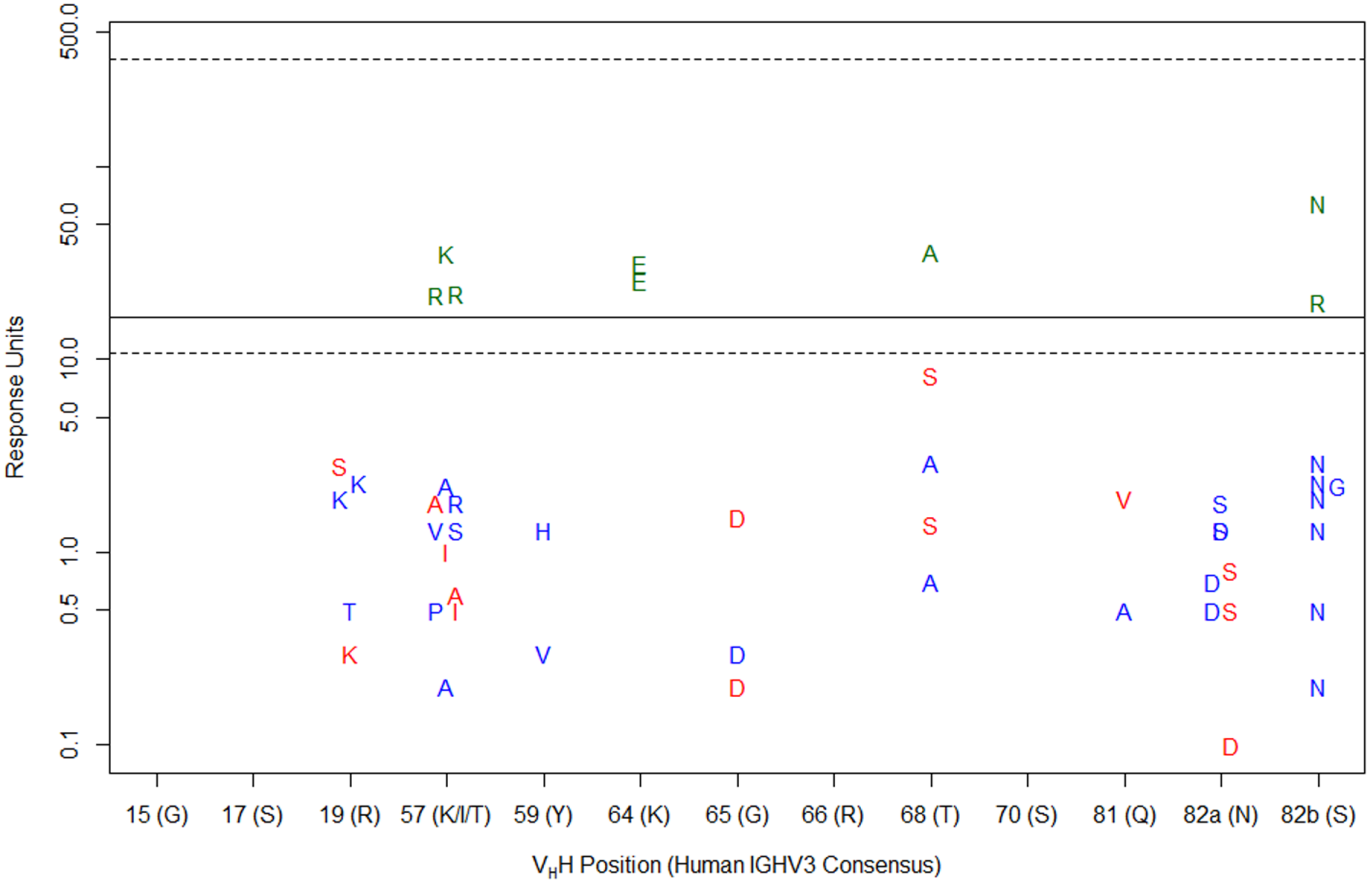
Identification of permissive and non-permissive residues for SpA binding at V_H_H SpA contact positions. 55 V_H_Hs of known sequence (FR sequences listed in S1 Table) were assayed for binding to immobilized SpA at a single concentration (250 nM) by SPR and the number of response units bound at the end of the injection was recorded. The solid line represents binding of HVH430 (17 RUs), an IGHV3-encoded human autonomous domain. For V_H_Hs bearing the human IGHV3 consensus residue (shown on the X-axis) at all 13 SpA contact positions, no data are plotted on the graph; instead, dotted lines are shown representing the 95% confidence interval (CI) for mean SpA binding of “wild-type” V_H_Hs bearing this consensus sequence. For V_H_Hs bearing single amino acid substitutions at any one of the 13 SpA contact positions, the relevant substitution is plotted on the graph in green (substitution tolerated) if SpA binding fell within the 95% CI for wild-type V_H_Hs (10–360 RUs), and red (substitution not tolerated) if it fell below. For V_H_Hs bearing multiple amino acid substitutions at SpA contact sites, substitutions are plotted on the graph in blue.

To investigate whether the conserved SpA-binding residues in human IGHV3 Abs were also important for V_H_H binding by SpA, we first determined the sequences of 55 V_H_Hs obtained in our lab along with an IGHV3-encoded human autonomous V_H_ domain, HVH430 [56], and assessed their binding to immobilized SpA at a single concentration (250 nM) by SPR (Fig 2; S1 Table). Many, but not all, camelid V_H_Hs shared the human IGHV3 consensus sequence at all thirteen SpA contact residues, suggesting that they may interact in a similar way with SpA. Complete or near-complete sequence conservation at some V_H_H positions (Gly15, Ser17, Arg66, Ser70 and Gln81) among both SpA-binding V_H_Hs and non-SpA-binding V_H_Hs prevented the assessment of their importance. In agreement with the conclusions of structural studies [25] and a previous mutagenesis study [57], the salt bridge formed between the Asp residue at position 36 of SpA domain D and the conserved Arg residue at V_H_H FR1 position 19 was indispensable, as its replacement with Lys, Ser or Thr abrogated SpA binding. Similarly, replacement of core Gly65 with negatively charged Asp, or of core Asn82a with Asp or Ser, had a destructive effect on SpA binding; the latter result is consistent with a previous mutagenesis study [57] which found that substitution of Asn82a with Ala abrogated SpA binding. Surprisingly, one V_H_H bearing a substitution of core Thr68 with Ala (V_H_H36) showed residual SpA binding. However, since V_H_Hs encoding Ala68 in combination with other substitutions did not bind SpA, and the more conservative replacement of Thr68 with Ser reduced or ablated SpA binding, we infer that that the limited polymorphisms tolerated at this position confer a partial destabilizing effect on SpA binding.

In agreement with previous work [18,23], we found that a variety of residues (Thr, Arg and Lys) were tolerated at the non-core V_H_H CDR2 position 57, although the full spectrum of tolerated residues could not be conclusively identified due to the co-occurrence of substitutions at other positions (Fig 2; S1 Table). Conversely, the presence of Ala57 clearly ablated SpA binding, and surprisingly, two V_H_Hs bearing Ile57, ICAM11-4 and V_H_H55, did not bind SpA; Ile57 is present in some germline human IGHV3 genes and was previously judged as permissive for SpA binding by some groups [23,25] but not others [18]. There was some indication that V_H_H Tyr59, which forms a H-bond with SpA Asp37 that was not considered a core interaction by Graille *et al*. [25], might be essential, as its substitution with His or Val (in combination with other substitutions in V_H_H26 and IGF1R-4) ablated SpA binding. Both reversal of charge at the non-core V_H_H position 64 (Lys→Glu) and substitution of non-core Ser82b with Asn or Arg appeared to be well tolerated, with no consistent detrimental effect on SpA binding.

Thus, the overall picture emerging from these data was that V_H_H interaction with SpA depended primarily on the same set of residues as human IGHV3 Abs, albeit with: (*i*) an unanticipated but minor degree of tolerance for variation at core position Thr68 and a broader tolerance at non-core positions Thr57, Lys64 and Ser82b; (*ii*) a potentially critical role for non-core V_H_H position Tyr59; and (*iii*) a destructive effect of Ile at V_H_H position 57, in contrast to the predictions of some previous studies. We attempted to corroborate these data using a set of V_H_H pentamers [35] but found that the multivalent nature of the pentamer:SpA interaction made it difficult to discriminate very weakly-binding from non-binding V_H_H pentamers (S1 Fig; S2 Table). We found no evidence to suggest that FR sequence polymorphisms outside the previously defined SpA interface [25] played any role in SpA binding, with one notable exception (V_H_H39): this V_H_H did not bind SpA despite bearing the human IGHV3 consensus sequence at all 13 SpA contact positions, but encodes a single-residue deletion at FR3 position 76. This provided the first preliminary evidence that FR3 positions 71–80 may influence structuring of nearby SpA contact residues and play an indirect role in modulating SpA binding.

### SpA binding is reliably conferred to V_H_Hs by humanization

The simplest possible strategy for rescuing SpA binding in non-SpA-binding V_H_Hs would be to revert any discrepancies at the 13 contact positions defined by Graille *et al*. [25] to the human IGHV3 consensus sequence. This is always accomplished, by definition, through the process of humanization, in which a llama V_H_H’s FRs are replaced almost entirely with those of the most closely homologous human IGHV3 gene. To confirm that this strategy would be successful, we took four *a priori* non-SpA-binding V_H_Hs (ICAM11-4 and ICAM34-1, directed against human ICAM-1; IGF1R-4 and IGF1R-5, directed against human IGF1R) and humanized them, thus reverting their FR sequences to human IGHV3 germline both at SpA contact positions (Table 1) and elsewhere (S3 Table). A fifth example, AFAI (a V_H_H directed against CEACAM6), was not humanized, but instead had two reversions incorporated *via* site-directed mutagenesis (Glu64Lys, Asp82bSer).

**Table 1:** FR sequences of five non-SpA-binding V_H_Hs at SpA contact positions.

V_H_H	V_H_H Position												
	15 (G)	17 (S)	19 (R)	57 (K/I/T)	59 (Y)	64 (K)	65 (G)	66 (R)	68 (T)	70 (S)	81 (Q)	82a (N)	82b (S)
ICAM11-4	G	S	R	I*	Y	K	G	R	T	S	Q	N	S
ICAM34-1	G	S	R	A*	Y	K	G	R	T	S	Q	N	G*
IGF1R-4	G	S	R	T	V*	K	D*	R	T	S	Q	N	S
IGF1R-5	G	S	R	A*	Y	K	G	R	T	S	Q	N	N*
AFAI	G	S	R	T	Y	E*	G	R	T	S	Q	N	D*

*Residues were reverted to the human IGHV3 germline consensus during humanization

SpA binding by these five camelid V_H_Hs was initially extremely weak or undetectable, as measured using single-concentration injections of 250 nM V_H_H over immobilized SpA in SPR. However, in each case, V_H_H humanization (or in the case of AFAI, reversion of both Glu64 to Lys and Asp82b to Ser) conferred SpA binding (S2 Fig) that was sufficient to enable their purification by SpA affinity chromatography (S3 Fig). In three of four cases, expression yields of the humanized V_H_Hs after IMAC purification were reduced by 50% or more compared to their wild-type llama counterparts (Table 2, left *vs*. middle columns), and in one case (ICAM11-4), the humanized V_H_H had a ~20-fold loss of affinity for its cognate antigen (Table 3, left *vs*. middle columns). Thus, perhaps unsurprisingly, SpA binding could be successfully introduced into five *a priori* non-SpA-binding V_H_Hs by humanization, although this came at the cost of reduced expression yield, and in one case, impaired antigen recognition.

**Table 2:** Expression yields (mg/L) in *E*. *coli* of wild-type llama V_H_Hs *vs*. their humanized or SpA-engineered counterparts.

V_H_H	Wild-type	Humanized	SpA-engineered
ICAM11-4	20.0	4.5	17.0
ICAM34-1	21.0	7.0	25.0
IGF1R-4	9.0	4.5	6.0
IGF1R-5	14.0	11.0	12.0
AFAI	6.0	n.d.	4.0

n.d., not determined

**Table 3:** Affinities for cognate antigen (pH 7.4, 25°C) of wild-type llama V_H_Hs *vs*. their humanized or SpA-engineered counterparts.

V_H_H	Wild-type	Humanized	SpA-engineered
ICAM11-4	0.9 nM	17 nM	1.3 nM
ICAM34-1	32 nM	26 nM	n.d.^a^
IGF1R-4	0.3 nM	0.4 nM	n.d.^a^
IGF1R-5	0.8 nM	0.9 nM	n.d.^a^
AFAI	+^b^	n.d.	+^b^

n.d., not determined

^a^Affinities were not determined on the assumption that since no loss of affinity was observed in the humanized V_H_H, SpA-engineered V_H_Hs (bearing fewer changes from wild-type) would also retain full affinity for antigen.

^b^Affinities were not determined using monomeric V_H_H; rather, binding of wild-type and SpA-engineered V_H_H pentamers are shown in S4 Fig.

### SpA binding can be conferred to V_H_Hs by limited site-directed mutagenesis without negatively impacting expression yield or antigen binding

To determine whether SpA binding could be restored in non-SpA binding V_H_Hs without incurring the negative consequences of humanization on expression yield and antigen binding, and to better understand the relative impacts of individual substitutions on SpA binding, we took the same five non-SpA binding V_H_Hs as above and reverted any discrepancies at the 13 SpA contact positions (Table 1) to the human IGHV3 consensus by site-directed mutagenesis. In one case (ICAM34-1), we also reverted an unusual Pro at position 75 to the human IGHV3 consensus Lys, based on the hypothesis that this Pro residue might affect structuring of surrounding SpA contact residues. Substitutions were incorporated either singly or in all possible combinations, and the SpA-binding affinities of the resulting engineered V_H_Hs were then determined by SPR.

In the case of ICAM11-4, a single substitution (Ile57Thr) was sufficient to restore 9.0 μM affinity for SpA (Table 4; in this table and hereafter, SpA-engineered V_H_Hs (underlined) are defined as the variants bearing the minimal substitutions from wild-type sequences required to restore SpA binding), near to the wild-type affinity range of 1–5 μM described for human IGHV3-encoded V_H_ domains [56]. In the case of ICAM34-1, two reversions (Ala57Thr and Pro75Lys) were necessary to recover any degree of SpA binding (9.6 μM), which was improved slightly by a third reversion (Gly82bSer, 4.5 μM). Likewise, two substitutions (Val59Tyr and Asp65Gly) were required to restore SpA binding (1.5 μM) to IGF1R-4, confirming the critical nature of the consensus residue at both of these positions for interaction with SpA. In the case of IGF1R-5, a single substitution (Ala57Thr) conferred 3.7 μM affinity for SpA, with no further affinity improvement obtained by reversion of position 82b from Asn to Ser. Finally, reversion of Asp82b to Ser in AFAI restored weak SpA binding (11.0 μM), which was further improved with the additional reversion of Glu64 to Lys (0.6 μM). This suggested a cumulative defect of both substitutions in the wild-type V_H_H, with Asp82b conferring more significant binding impairment than Glu64.

**Table 4:** Affinities for SpA (pH 7.4, 25°C) of wild-type llama V_H_Hs *vs*. their SpA-engineered counterparts.

V_H_H	Wild-type	SpA-engineered (K_D_)
ICAM11-4	n.b.	I57T (9.0 μM)*
ICAM34-1	n.b.	A57T (n.b.)
		P75K (n.b.)
		G82bS (n.b.)
		A57T, P75K (9.6 μM)
		A57T, G82bS (n.b.)
		P75K, G82bS (n.b.)
		A57T, P75K, G82bS (4.5 μM)*
IGF1R-4	n.b.	V59Y (n.b.)
		D65G (n.b.)
		V59Y, D65G (1.5 μM)*
IGF1R-5	n.b.	A57T (3.7 μM)*
		N82bS (n.b.)
		A57T, N82bS (3.5 μM)
AFAI	n.b.	E64K (n.b.)
		D82bS (11.0 μM)
		E64K, D82bS (0.6 μM)*

n.b., no binding

*SpA-engineered V_H_Hs (defined as the variants bearing the minimal substitutions from wild-type sequences required to restore SpA binding) are underlined

In summary, at least in the five examples above, SpA binding could be conferred to non-SpA binding V_H_Hs using limited numbers (1–3) of amino acid substitutions. The resulting engineered V_H_Hs bound SpA with affinities similar to those of human IGHV3-encoded Fabs, could be purified by SpA affinity chromatography (S3 Fig), and had expression yields after IMAC purification that were indistinguishable from their non-SpA binding counterparts (Table 2, left *vs*. rightmost column). In the case of ICAM11-4, the engineered SpA-binding V_H_H retained full affinity for ICAM-1, unlike the humanized V_H_H (Table 3, middle *vs*. right columns). The data provided from these mutagenesis studies also expanded our understanding of the spectrum of permissive and non-permissive V_H_H residues for SpA binding, which are listed in Table 5, and clearly supported the hypothesis that residues outside the SpA contact interface such as Pro75 can indirectly modulate SpA binding.

**Table 5:** Spectrum of residues tolerated at V_H_H SpA contact positions, as demonstrated by experimental data or as predicted by *in silico* mutagenesis.

V_H_H Position	Experimentally Validated		Computationally Predicted	
	Tolerated	Not tolerated	Tolerated	Not tolerated
15 (G)	G, D^a^	-	G	All others
17 (S)	S, A^a^	-	Many	-
19 (R)	R	K, S, A^a^, Q^a^	R	All others
57 (K/I/T)	K, R, T	A, I	K, R, T	All others
59 (Y)	Y	V	Y	All others
64 (K)	K (E)*	-	Many	-
65 (G)	G	D	G	All others
66 (R)	R	-	R	All others
68 (T)	(A)	S	I, M, V	Many
70 (S)	S	-	Many	I, T, V
75 (K)	A, E, K, Q, R	P	Many	I, T, V
81 (Q)	Q	-	M, R	Many
82a (N)	N	D, S, A^a^	I, L, M, V	Many
82b (S)	S, N (G)	D	Many	Many

Residues in brackets are tolerated but result in some loss of binding

^a^Data from Fridy *et al*. [57]

### 3.4. *In silico* modeling of the SpA:V_H_H interaction by virtual mutagenesis

**Fig 3:**
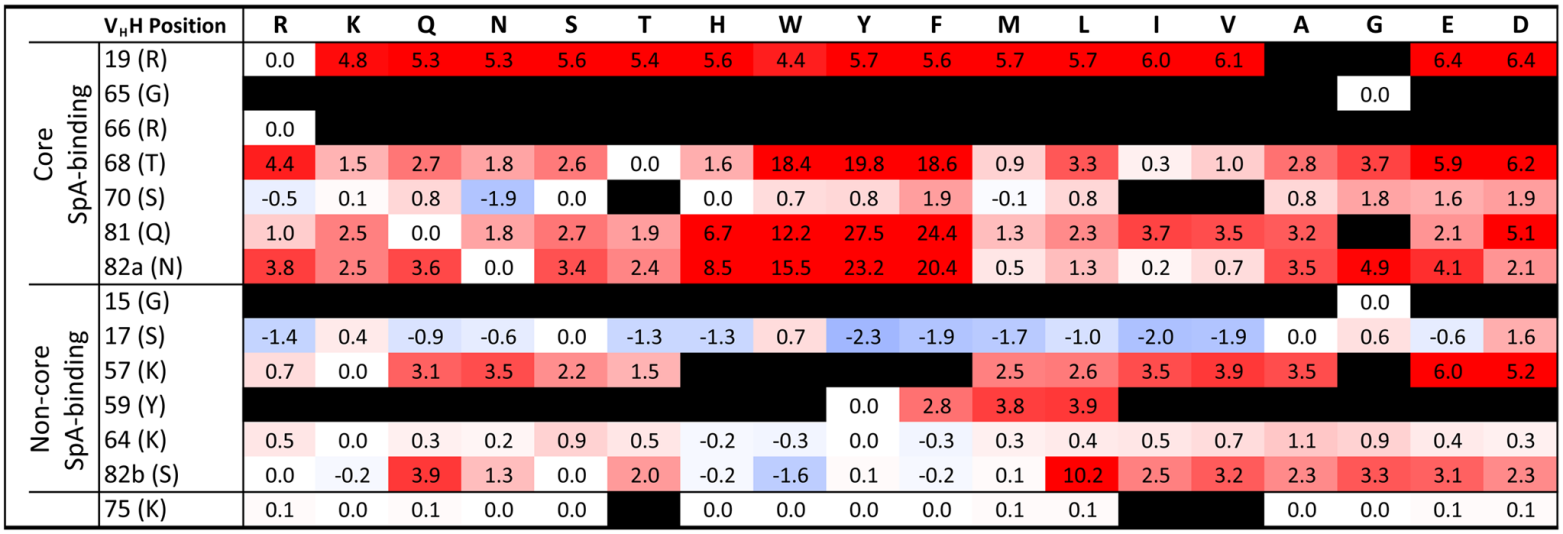
Effect of *in silico* scanning mutagenesis of V_H_H SpA contact residues on V_H_H folding and SpA binding. Consensus Z-scores for SpA binding were calculated over the SIE-SCWRL, FoldX and Rosetta protocols for mutations to 18 amino acids (shown at the top) at 14 V_H_H positions (shown on the left). Predicted change to ΔG (free energy of binding, kCal/mol) for SpA binding to V_H_H are shown numerically and colored in shades of red or blue for single-point mutations predicted to strengthen or weaken SpA binding, respectively, relative to the wild-type amino acid corresponding to the human IGHV3 sequence. Mutations predicted by FoldX to destabilize V_H_H folding by more than 10-fold relative to parental IGHV3 sequence are highlighted in black shading. See Materials and Methods and Sulea *et al*. [50] for further details.

To support and extend the experimental results above, we carried out a computational assessment of the effects of virtual mutagenesis of V_H_H SpA contact residues on SpA binding (Fig 3 and Table 5). We used a consensus approach for mutant building and scoring that has been found to afford improved ranking of Ab:antigen binding affinities relative to various individual methods when applied to over 200 single-point antibody mutants of the SiPMAB database curated from the literature [50]. We used the published SpA:IGHV3 Fab structure [25] as the basis for *in silico* analyses, but prior to virtual mutagenesis excluded the light chain from consideration and “camelized” the human IGHV3 domain by substitution of FR2 Val37Phe, Gly44Glu, Trp47Ala and Leu45Arg. The results of *in silico* mutagenesis provided an explanation for the almost complete conservation of Gly15, core Gly65 and core Arg66 in V_H_Hs, since substitution of these residues was predicted to drastically destabilize V_H_H folding; the backbone phi-psi dihedral angles of Gly15 and Gly65 are (92, -18) and (92, -20), respectively, in the 4NC0 crystal structure and are high-energy regions for non-glycine amino acids. Conversely, Ser17 and core Ser70 were predicted to tolerate substitution with a variety of residues despite being almost totally conserved in V_H_Hs. In the case of core V_H_H Gly65, experimental evidence contradicted computational predictions of fold destabilization by substitution with Asp, as this residue was observed in three V_H_H monomers and one V_H_H pentamer (S1 and S2 Tables). In agreement with experimental data, core V_H_H Arg19 was critical for interaction with SpA, and could not be substituted even conservatively by Lys. Also in line with experimental data, moderate but potentially non-destructive reductions of SpA binding were predicted when V_H_H core Thr68 was substituted with Ser, Ala and several other residues; similar trends were observed for core Gln81 and Asn82a. Virtual mutagenesis predicted a critical role for non-core V_H_H positions 57 (Thr, Lys, and Arg) and Tyr59, a negligible role for non-core V_H_H position Lys64 and variable effects of Ser82b substitution, with minor loss of binding resulting from Asn substitution and more pronounced defects resulting from substitution with Gly or Asp. Interestingly, V_H_H position 75 was predicted to tolerate substitution with most residues except those with beta-branched side chains, which probably disrupt the H-bonded turn of the FR3 loop at this location. We speculate that the effect of Pro substitution at this position has a similar effect and the incurred local misfolding may be propagated to adjacent SpA contact residues.

Overall, the experimental and computational data were congruent and together supported the classification of SpA contact residues of V_H_Hs into three categories: (*i*) critical for folding (Gly15, Gly65, Arg66) and/or binding (Arg19, Tyr59, Gly65); (*ii*) partially permissive to specific substitutions (Thr68, Gln81, Asn82a, Thr/Lys/Arg57, Ser82b) and generally tolerant to many substitutions (Ser17, Ser70, Lys64). For V_H_H residues falling into the second and third categories, however, the spectrum of amino acids predicted computationally to be tolerated at each position could not be validated experimentally using the available data.

### Next-generation DNA sequencing of camelid V_H_H repertoires suggests that species differences in SpA binding may arise from differential frequency of Ile57 polymorphism

SpA binding is observed rarely in V_H_Hs of dromedary origin and more commonly in those of llama origin, although a detailed comparison of the frequency of SpA binding by species has yet to be published [30]. To identify potential explanations at the level of protein sequences that might account for this observation, we used next-generation sequencing technology to interrogate four V_H_H repertoires to variable depths (0.4–6.0×10^5^ reads; S4 Table). The source of the V_H_H repertoires were either lymphocytes derived from three individual llamas or a single pooled sample of alpaca, camel and llama lymphocytes [34].

**Fig 4:**
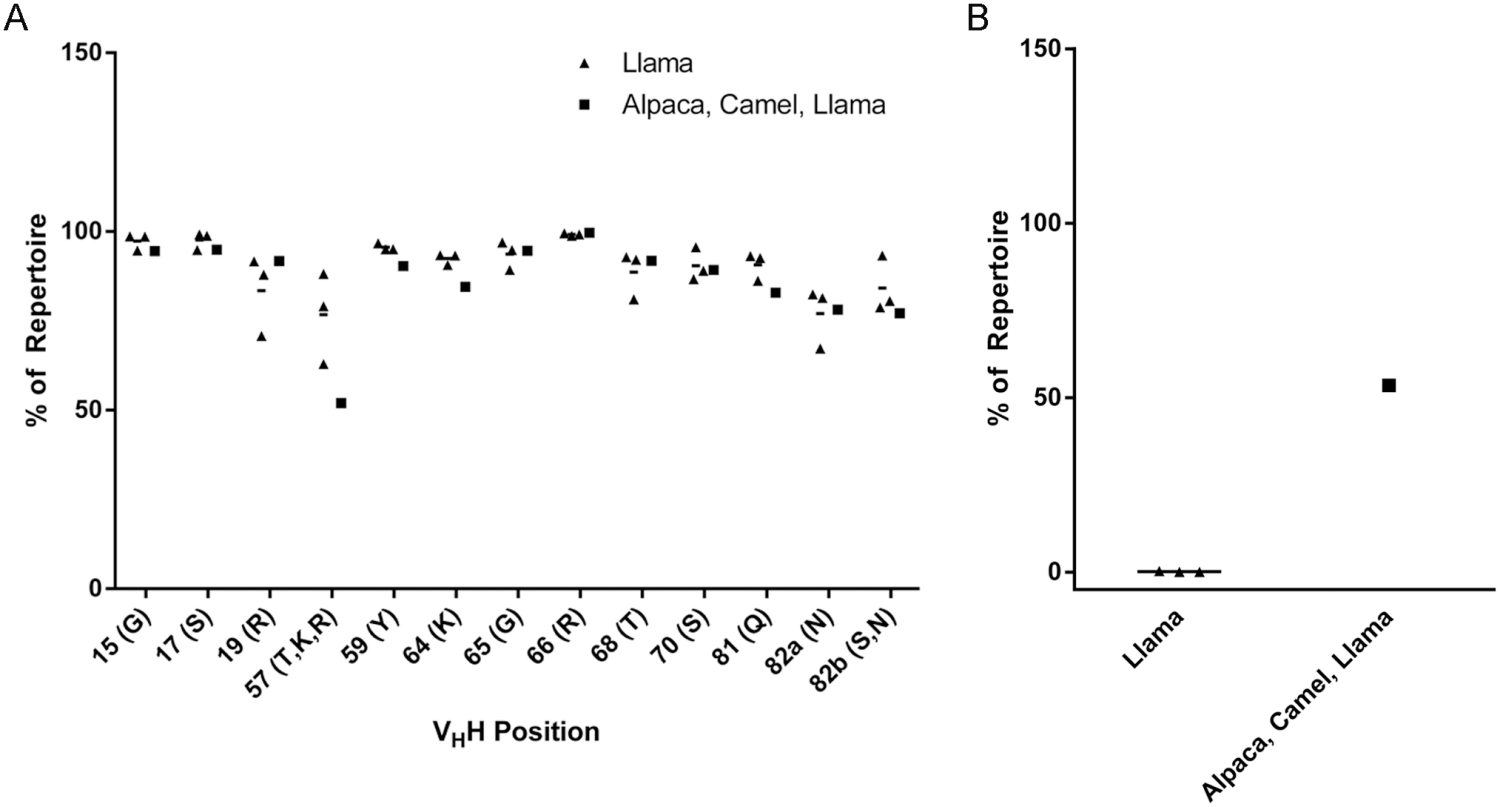
Next-generation DNA sequencing of llama and mixed-species (alpaca, camel, llama) V_H_H repertoires and analysis of SpA contact residues. **(A)** Frequency of SpA-permissive residues at contact positions in llama *vs*. mixed-species V_H_H repertoires. Triangles represent individual llama repertoires, squares represent a single pooled mixed-species repertoire, and horizontal lines represent the mean. Residues permissive for SpA binding are indicated on the X-axis. **(B)** Frequency of putative CDR1-CDR3 intradomain disulfide bond in llama *vs*. mixed-species V_H_H repertoires. Horizontal lines represent the averages.

As shown in Fig 4A, there was no general defect in SpA binding evident from the sequences of V_H_H repertoires of camels and alpacas. The proportion of V_H_H sequences bearing the human IGHV3 consensus sequence at SpA contact positions was broadly similar in all four repertoires, except at position 57, where Ile was much more frequently present in dromedary and/or alpaca V_H_Hs (llama repertoire: ~5% vs. mixed llama, alpaca and camel repertoires: ~22%). A major difference was observed in the frequency of putative CDR1-CDR3 intradomain disulfide bonds, as indicated by the simultaneous presence of Cys residues at any position within both these regions, which were almost entirely absent in the repertoires of llamas but very frequent in the mixed-species repertoire (Fig 4B). To confirm that CDR1-CDR3 disulfide bridging had no effect on SpA binding, we ablated this disulfide from a non-SpA-binding dromedary V_H_H as well as introduced it into the engineered SpA-binding variant of the same V_H_H (Ile57Thr; S5 Table). Neither introduction nor ablation of the CDR1-CDR3 disulfide bridge had any impact on SpA binding, at least in the case of the single dromedary V_H_H tested here.

## Discussion

SpA affinity chromatography has become the purification method of choice for most antibody manufacturers over the last 15 years, especially in the therapeutic Ab pipeline [8]. For many applications, V_H_Hs are fused to mouse or human F_c_ regions for mammalian cell production and easily purified using conventional methods [58]. However, in some circumstances (*e*.*g*., *in vivo* imaging [59]; co-crystallization [60]; tumor targeting requiring tissue penetration [61]), the small size of the V_H_H molecule is essential, and the addition of affinity tags for purification may detract from the stability and homogeneity of the final product. The same is true of V_H_H pentamers, and the starting point for this work was the observation that His- and/or Myc-tagged pentamers had increased propensity for aggregation and non-specific binding than their untagged counterparts. Thus, tagless strategies for V_H_H purification are highly desirable.

Here, we present a detailed description of the sequence features of SpA-binding and SpA-non-binding V_H_Hs, and by extension, the features governing the interaction between SpA and other immunoglobulin V_H_ domains. Our results have important distinctions from those inferred from the SpA:Fab crystal structure [25]. First, we found that several core positions in the V_H_H:SpA interface can tolerate a limited degree of polymorphism without ablating SpA binding (shown experimentally: Thr68; predicted computationally: Thr68, Ser70, Gln18, Asn82a). Second, on the basis of both experimental data and computational structural modeling, we found that non-core V_H_H Thr/Lys/Arg57 and Tyr59 are indispensable for SpA binding, and that Ile57 had a destructive effect on SpA binding. Third, we found that V_H_H Pro75 exerts a destabilizing effect on SpA binding, potentially by altering the conformation of FR3 sufficiently to displace SpA contact residues some distance away. Given this surprising finding, a potential role for additional V_H_H polymorphisms outside the SpA interface in determining SpA binding cannot be ruled out. However, the fact that a three amino acid insertion in the FR3 of V_H_H52 (S1 Table) did not impair SpA binding suggests that epistatic effects involving “action at a distance” may be rare.

In the analyses presented here, we did not attempt to rank the relative effects of individual V_H_H substitutions on SpA binding, instead opting to categorize them dichotomously as permissive or non-permissive for SpA binding (Table 5). The rationale for this decision was two-fold. First, several substitutions were observed experimentally in only a single V_H_H, and their effects may depend on the sequence background and presence of other polymorphisms. Second, the weak overall affinity of the SpA:V_H_H interaction makes determinations of monovalent binding strength challenging below a certain threshold, and weak residual binding is difficult to rule out experimentally; this was clearly evident in comparisons of SpA binding of V_H_H monomers and pentamers bearing similar sequences. Nevertheless, the overall consistency between experimental data and computational predictions, as well as with the limited mutagenesis data of Fridy *et al*. [57], provides a strong degree of confidence in the general effects of many of the substitutions described here. We caution, however, that computational predictions of minor or moderate reductions in SpA binding were not always accurate in the degree of their effects, and that the safest course is to revert all V_H_H contact positions back to the human IGHV3 consensus, even if some of the original polymorphisms might have been tolerated.

Using next-generation DNA sequencing of llama, alpaca and dromedary V_H_H repertoires, we found that the most likely explanation for non-SpA binding of dromedary V_H_Hs was the presence of non-permissive residues at SpA contact positions, especially Ile57. Since the comparison in our analysis was between llama and mixed-species repertoires, the frequency of deleterious polymorphisms detrimental to SpA binding, including Ile57, is likely even higher than shown here in the repertoires of alpacas and/or dromedaries. It remains unclear whether such differences arise through germline polymorphism or somatic mutation, although comparison of germline [26] and rearranged [27] camelid V_H_H sequences favours the latter hypothesis. Also unclear are the reasons why Ile57 should be a hotspot for mutation in dromedaries and/or alpacas, but not llamas.

On the basis of these data, we propose the following general strategy for conferring SpA binding upon camelid V_H_Hs: (*i*) ensure that FR1 residues Gly15, Ser17 and Arg19 are present and revert any discrepancies to this consensus; (*ii*) ensure that CDR2 residue Thr/Lys/Arg57 is present and revert any discrepancies to this consensus; (*iii*) ensure that FR3 residues Tyr59, Lys64, Gly65, Arg66, Thr68, Ser70, Gln81, Asn82a, Ser/Asn82b are present and revert any discrepancies to this consensus; and (*iv*) closely examine FR3 positions 71–80 for Pro residues (especially at position 75) and unusual deletions and revert these to the nearest human IGHV3 germline residue. We have found no evidence to suggest that any of the substitutions introduced following these rules affect the expression yield, solubility, stability or aggregation status of V_H_Hs, and while some may not be essential for SpA binding, they are also not harmful. While there were no affinity penalties resulting from these substitutions for any of the five V_H_Hs shown here, the necessity of restricting CDR2 position 57 diversity to Thr, Lys or Arg may compromise the affinity of other V_H_Hs.

In conclusion, we have identified the sequence hallmarks responsible for determining camelid V_H_H binding by SpA, which provide an explanation for species differences in V_H_H SpA reactivity. We used this information to develop a strategy for engineering V_H_Hs to introduce SpA binding and enable their tagless purification by SpA chromatography. This strategy may also apply to Ab fragments of other species, or at least those that share homology with human IGHV3 Abs.

## Supporting Information

S1 Fig Binding of V_H_H pentamers at 100 nM to immobilized SpA by SPR. Each pentamer (FR sequences listed in S2 Table) was injected for 2 min and the number of response units bound at the end of the injection was measured. For pentamers bearing the human IGHV3 consensus residue at all 13 SpA contact positions, no residues are plotted on the graph; instead, dotted lines are shown representing the 95% confidence interval (CI) for mean SpA binding of wild-type pentamers bearing this consensus sequence. For pentamers bearing single amino acid substitutions at any one of the 13 SpA contact positions, the relevant substitution is plotted on the graph in green (substitution tolerated) if SpA binding fell within the 95% CI for wild-type pentamers, and red (substitution not tolerated) if not. For pentamers bearing multiple amino acid substitutions at SpA contact sites, substitutions are plotted on the graph in blue. We used a verotoxin B-irrelevant peptide fusion as a negative control to rule out potential interactions between SpA and the pentamerization domain (data not shown).
(TIF)

S2 Fig SpA binding by SPR of four llama V_H_H monomers (ICAM11-4, ICAM34-1, IGF1R-4 and IGF1R-5; dotted lines) and one llama V_H_H pentamer (AFAI; dotted line) along with their humanized counterparts (solid line). V_H_H monomers and pentamers (250 nM) were injected over immobilized SpA for 2 min and allowed to dissociate as described in methods.
(TIF)

S3 Fig Representative chromatogram overlay of purification of llama, humanized and SpA-engineered ICAM11-4 V_H_H. All three V_H_Hs were produced in *E*. *coli* TG1 cells in 1L 2×YT overnight cultures grown at 37°C. V_H_Hs were extracted from periplasmic space by osmotic shock and purified using a HiTrap Protein A HP column on an ÄKTA FPLC protein purification system (GE Healthcare).
(TIF)

S4 Fig Binding of wild-type or SpA-engineered AFAI V_H_H pentamers at either 1 nM (A) or 50 nM (B) to immobilized CEACAM6 N-terminal domain by SPR. V_H_H pentamers were injected over immobilized CEACAM6 N-terminal domain for 3 min and allowed to dissociate as described in methods.
(TIF)

S1 Table FR sequences of SpA-binding and non-SpA-binding V_H_H monomers. (DOCX)

S2 Table FR sequences of SpA-binding and non-SpA-binding V_H_H pentamers. (DOCX)

S3 Table FR sequences of five non-SpA-binding V_H_Hs and their humanized SpA-binding counterparts. (DOCX)

S4 Table Metrics for Illumina MiSeq NGS data used in this study. (DOCX)

S5 Table FR and CDR sequences of a SpA-binding (Thr57) and non-SpA-binding (Ile57) dromedary V_H_H and effect of CDR1-CDR3 disulfide bridge on SpA binding. (DOCX)
