# Supplementary figures and images for: A Rational Engineering Strategy for Designing Protein A-Binding Camelid Single-Domain Antibodies
Source: PLoS One. 2016 Sep 15;11(9):e0163113. doi: 10.1371/journal.pone.0163113 (PMC5025174; doi:10.1371/journal.pone.0163113)

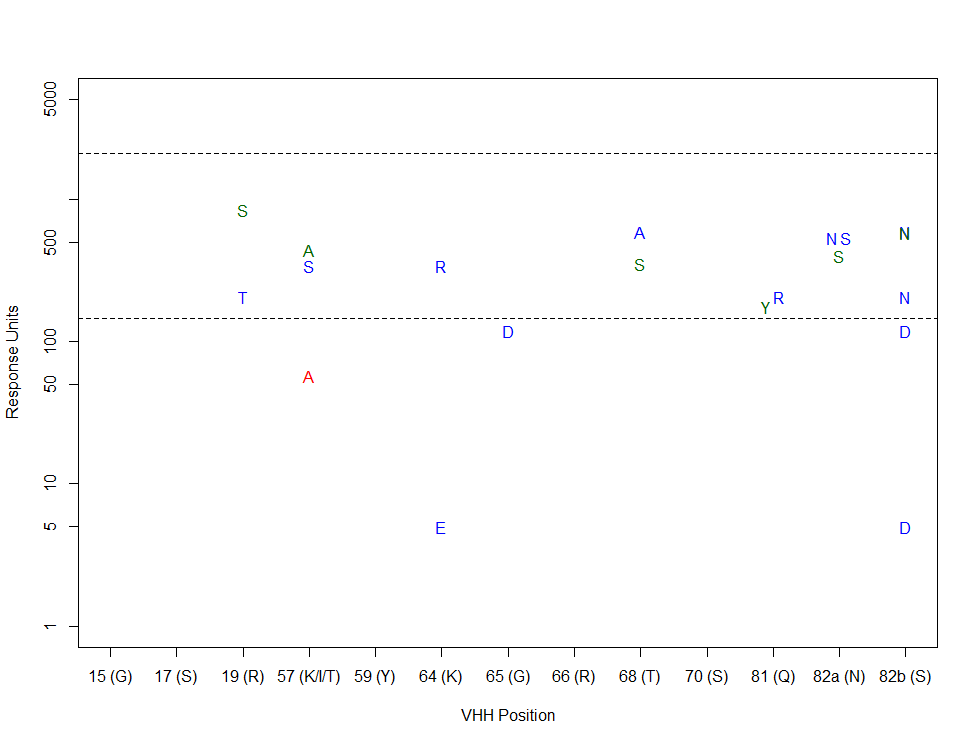

Supplement: S1 Fig — Each pentamer (FR sequences listed in S2 Table) was injected for 2 min and the number of response units bound at the end of the injection was measured. For pentamers bearing the human IGHV3 consensus residue at all 13 SpA contact positions, no residues are plotted on the graph; instead, dotted lines are shown representing the 95% confidence interval (CI) for mean SpA binding of wild-type pentamers bearing this consensus sequence. For pentamers bearing single amino acid substitutions at any one of the 13 SpA contact positions, the relevant substitution is plotted on the graph in green (substitution tolerated) if SpA binding fell within the 95% CI for wild-type pentamers, and red (substitution not tolerated) if not. For pentamers bearing multiple amino acid substitutions at SpA contact sites, substitutions are plotted on the graph in blue. We used a verotoxin B-irrelevant peptide fusion as a negative control to rule out potential interactions between SpA and the pentamerization domain (data not shown). (TIF) [file pone.0163113.s001.tif]

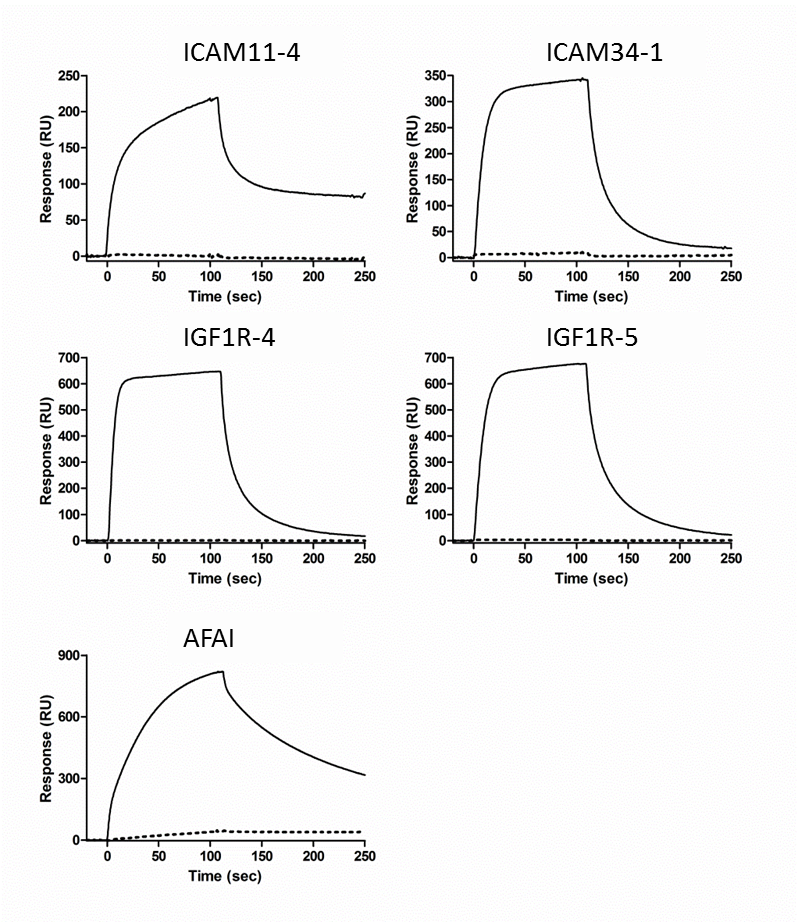

Supplement: S2 Fig — VHH monomers and pentamers (250 nM) were injected over immobilized SpA for 2 min and allowed to dissociate as described in methods. (TIF) [file pone.0163113.s002.tif]

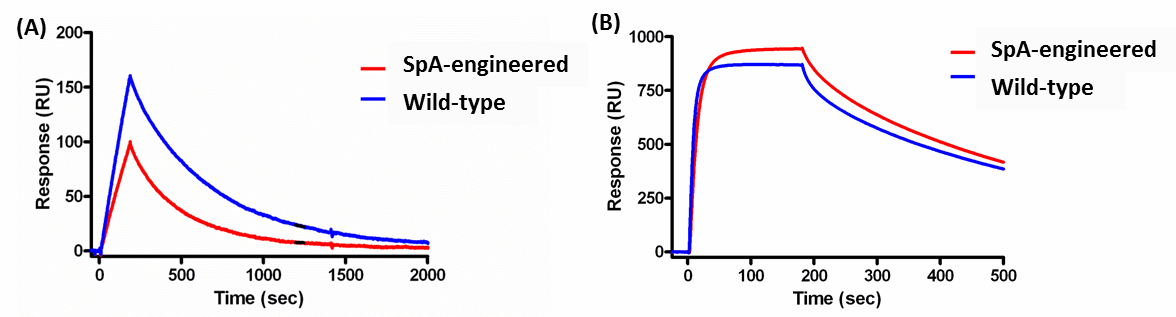

Supplement: S4 Fig — VHH pentamers were injected over immobilized CEACAM6 N-terminal domain for 3 min and allowed to dissociate as described in methods. (TIF) [file pone.0163113.s004.tif]
