# Supplementary material for: A Rational Engineering Strategy for Designing Protein A-Binding Camelid Single-Domain Antibodies
Source: PLoS One. 2016 Sep 15;11(9):e0163113. doi: 10.1371/journal.pone.0163113 (PMC5025174; doi:10.1371/journal.pone.0163113)
Supplement: S1 Table — (DOCX) [file pone.0163113.s005.docx]

**S1 Table**. FR sequences of SpA-binding and non-SpA-binding V_H_H monomers.

| **V_H_H** | **Disulfide Bonds** | **FR1** | **FR2** | **CDR2** | **FR3** | **FR4** | **SpA Binding (RUs bound at end of injection)** |
| --- | --- | --- | --- | --- | --- | --- | --- |
| HVH430 | CDR3-CDR3 | QVQLVESGGGLIKPGGSLRLSCAAS | MSWVRQAPGKGLEWVSA | T | YYADSVKGRFTISRDNSKNTVYLQMNSLRAEDTAVYYC | WGQGTMVTVSS | 16.6 |
| VHH1 | - | QVQLVESGGGLVQPGGSLRLACTAS | MGWFRQTPGKERDFVAS | T | HYEDSVKGRFTISRDNAKKAVYLQMNSLKPEDTAVYYC | WGQGIQVTVSS | 24.5 |
| VHH2 | - | QVQLVESGGGLVQAGGSLSLSCAAS | MGWFRQAPGKEREFVAR | T | SYADSVKGRFTISRDNDKNTLYLQMNSLKPEDTAVYYC | WGQGTQVTVSS | 2.8 |
| VHH3 | - | QVQLVESGGGLAQAGGSLRLSCAAS | MGWFRQAPGQERETVAA | T | YYADSVKGRFTISGDYAKNTVFLVMNSLKPEDTAVYYC | WGQGTQVTVSS | 1.9 |
| VHH4 | - | QVQLVESGGGLVQPGGSLRLSCAAS | MGWYRQAPGKQRELVAS | T | YYEDFVKGRFTISRDNAKNTVALQMDNLKPEDTAVYYC | WGQGTQVTVSS | 1.3 |
| VHH5 | - | QVQLVESGGGWVQAGGSLRLSCAAS | MGWFRQAPGKEREFVAA | R | YYADSVKGRFTISMDNAKNTVYLQMSSLKPEDTAVYYC | WGQGTQVTVSS | 1.8 |
| VHH6 | - | QVKLEESGGGLAQAGGSLRLSCAAS | MGWFRQAPGKEREFVAM | T | NYADSVKGRFTISRDNAKNTVDLQMNSLTPEDSAVYYC | WGQGTQVTVSS | 192 |
| VHH7 | - | QVQLVESGGGLVQPGGSLRLSCAAS | MGWYRQAPGKQRELVAT | T | NYADSVKGRFTISTDNTKNTVYLQMNSLKPEDTAVYYC | WGQGTQVTVSS | 194 |
| VHH8 | - | QVQLVESGGGLVQPGGSLRLSCAAS | MGWHRQAPGKQREFVAR | T | RYADSVEGRFTISRDNAKNTVYLQMNSLKPEDTAVYYC | WGQGTQVTVSS | 31.5 |
| VHH9 | - | QVQLVESGGGLVQAGDSLRLSCAAS | MGWFRQAPGKEREFVAA | T | LYEDSAKGRFTISRDNAKNAVYLQMNSLKPEDTAVYYC | WGQGTQVTVSS | 18.4 |
| VHH10 | - | QVQLVESGGGLVQPGGSLRLSCAAS | MGWYRQAPGKERELVAN | T | KYADSVKDRFTISRDNAQNTIYLQMNSLKPEDTAVYYC | WGQGTQVTVSS | 1.5 |
| VHH11 | - | QVQLVESGGGLVQAGGSLRLSCAAS | MGWFRQAPGKEREFVAA | T | RYADSVKGRFTISRDNAKNMVYLQMNRVKPEDTAVYYC | WGQGTQVTVSS | 19.8 |
| VHH12 | - | QVQLVESGGGLVQPGGSLRLSCAAS | GSWYRQAPGKQREVVAR | T | NYADFVEGRFTISQDTAKKTLYLQMNSLKPEDTAVYYC | WGQGIQVTVSS | 25.6 |
| VHH13 | - | QVQLVESGGGLVQAGASLRLSCAAS | MGWFRQAPGKEREFVAG | T | FYADSVKGRFTISRDNAKNTMYLQMSSLKPEDTAVYYC | WGKGTQVTVSS | 0.8 |
| VHH14 | - | QVQLVESGGGLVQVGGSLRLSCAAS | MGWFRQAPGKEREFVAA | T | YYADSVKGRFTISRDNAKNTVYLQMNSLKPEDTAIYYC | WGQGTQVTVSS | 316 |
| VHH15 | - | QVELVESGGGLVQAGGSLRLSCAAS | MGWFRQAPGKEREFVAT | R | YYADSVKGRFTISRDNAKNTVFLQMNSLKPEDTAVYYC | WGQGTQVTVSS | 21.9 |
| VHH16 | - | QVQLVESGGGLVQAGGSLRLSCAAS | MGWFRQAPGKEREFVAA | T | VYVDSVKGRFTISRDNAKNTVYLQMSSLKPEDTAVYYC | WGQGTQVTVSS | 0.5 |
| VHH17 | - | QVQLVESGGGLVQAGGSLRLSCAAS | MGWFRQAPGKEREFVAT | T | YYGDSVKGRFTISRDNAKNSVYLQMNSLKPEDTAVYYC | WGQGTQVTVSS | 97.6 |
| VHH18 | - | QVQLVESGGGLVQPGGSLRLSCSVS | LAWHRQAPGKEREWVAL | T | NYANSVKGRFTISRDNSKNTVYLQMNSLKPEDTAVYYC | WGQGTQVTVSS | 107 |
| VHH19 | - | QVQLVESGGGLVQPGGSLKLSCAVS | VSWYRQAPGKQREFVAV | T | TYKDSVKGRFTISRDNTRNTAYLQMNNLKPEDTAVYYC | WGQGTQVTVSS | 1.9 |
| VHH20 | - | QVQLVESGGGLVQPGGSLRLSCTAS | VSWYRQAPGKEREFVAV | T | TYKDSVKGRFTISRDNTRNTGYLQMNNLKPEDTAVYYC | WGQGTQVTVSS | 64.5 |
| VHH21 | - | QVQLVESGGGLVQPGGSLRLSCAAS | MTWYRQAPGEQREFVAI | T | KYSDSVKGRFSISIDNAKNTVYLQMNSLKAEDTAVYYC | WGQGTQVTVSS | 1.4 |
| VHH22 | - | QVQLVESGGGLVQPGGSLRLSCAAS | MTWYRQAPGDEREFVAI | T | KYADSVKGRFSISIDNAKNTVYLQMNSLKAEDTAVYYC | WGQGTQVTVSS | 8.2 |
| VHH23 | - | QVQLVESGGALVQPGGSLRLSCSAS | LAWHRQAPGKEREWVAL | T | KYAGSVKGRFTISRDNDKNTVYLQMDSLKPEDTAVYYC | WGQGTQVTVSS | 0.1 |
| VHH24 | - | QVKLEESGGGLVQPGGSLKLSCAVS | VSWYRQAPGKQREFVAV | T | TYKDSVKGRFTISRDNTRNTAYLQMNNLKPEDTAVYYC | WGQGTQVTVSS | 2.3 |
| VHH25 | FR2-CDR3 | QVQLVESGGGLVQPGGSLTLSCVVS | VGWSRRVPGKEREAVSC | P | WYQDSVKGRFTVSKDNARNTVYLAMDNLKPEDTAVYYC | WGQGTQVTVSS | 0.5 |
| VHH26 | - | EVQLVESGGGLVQPGGSLRLSCTVS | MGWHRQAPGKQREMVAG | S | NHAESVKGRFTISRDNSKNTVYLQMNNLKPEDTAVYYC | WGQGTQVTVSS | 1.3 |
| VHH27 | - | QVQLVESGGGLVQAGGSLKLSCAAS | MAWFRQAPGKEREFVTR | T | HYSDSVKGRFTISRDNAENTVFLQMNSLNAEDTAVYYC | WGQGTQVTVSS | 0.3 |
| VHH28 | - | QVQLVESGGGLVQTGGSLRLSCGIS | IGWYRQAPGKQRELVAA | R | KYADSVKGRFTISRDNAKNTVYLQMNSLKPEDTAVYYC | WGQGTQVTVSS | 21.6 |
| VHH29 | - | QVQLVESGGGLVQAGGSVRLSCAAS | MGWFRQAPGKERESVAA | T | YYTDSAKGRFTISRDNAKNTVYLQMNSLKPEDTAVYYC | WGQGTQVTVSS | 30.3 |
| VHH30 | - | QVQLVESGGGVVQPGGSLRLSCAAS | MSWYRQAPGKQRELVAA | T | NYADSVKGRFTISRDNAKNTVYLQMNSLRPEDTAVYFC | WGQGTQVTVSS | 121 |
| VHH31 | - | QVQLAESGGGLVQPGGSLRLSCVIS | MGWYRQAPGKRREQVAL | T | NYVDSVKGRFTISRDNARNTMYLQMNSLKPEDTAVYYC | WGQGTQVTVSS | 18.9 |
| VHH32 | - | QVQLVESGGGLVQAGGSLRLSCAAS | MGWFRQAPGKEREFVAS | T | DYADSVKGRFAISIDNAKNTVYLQMNNLKFEDTAVYYC | WGQGTQVTVSS | 2.9 |
| VHH33 | - | QVQLVESGGGLVQAGGSLRLSCAAS | MGWFRQGPGKERDFVAS | T | DYADSVKGRFAISRDNAKNTVYLQMDSLKPEDTAVYFC | WGQGTQVTVSS | 0.7 |
| VHH34 | - | QAQHVESGGGLVQPGGSLRLSCAAS | MGWFRQAPGKRRELVAD | T | TYADSVKGRFTISRDNAKNTVYLQMNSLKPEDTAVYYC | WGQGTQVTVSS | 158 |
| VHH35 | - | QVQLVESGGGLVQAGGSLRLSCAAS | MGWFRQAPGKEREFVAA | T | VYADSVKDRFTISRDNAKNTVFLQMNSLKPEDTAVYYC | WGQGTQVTVSS | 0.2 |
| VHH36 | - | QVQLVESGGGLVQAGGSLRLSCAAS | MGWFRQAPGKEREFVAA | T | RYGDSVKGRFAISRENAKNTMYLQMNSLKPEDTAIYYC | WGQGTQVTVSS | 35.6 |
| VHH37 | - | QVKLEESGGGLVQAGDSLRLSCAAS | MGWYRQAPGKQRERVAV | T | SYIDSVKGRFTISRDNAKNTVYLQMNSLKPEDTAVYYC | RGQGTQVTVSS | 800* |
| VHH38 | - | QVKLEESGGGLVQAGGSLRLSCAAS | MGWFRQAPGKEREFVAG | T | HYAYSVKGRFTISRDNAANTVELQMNSLKPEDTAVYFC | WGQGTQVTVSS | 420* |
| VHH39 | FR2-CDR3 | EVQLQASGGGLVQPGGSLRLSCAAS | IGWFRQAPGKEREGVSC | T | YYADSVKGRFTISRHNAK-TVYLQMNSLKPEDTAVYYC | WGQGTQVTVSS | 3.5 |
| VHH40 | FR2-CDR3 | EVQLQASGGGLVQPGGSLRLSCAAS | IGWFRQAPGKAREGVAC | A | DYTDSVKGRFTISRDIARNTVYLQMNSLKPEDTAVYYC | WGQGTQVTVSS | 0.6 |
| VHH41 | - | QVKLEESGGGLVQAGGSLRLSCAAS | MGWFRQAPGKEREFVAA | T | YYADSVKGRFTISRDNAKNTVYLQMNSLKPEDTAVYYC | WGQGTQVTVSS | 81.0 |
| VHH42 | - | QVQLVESGGGLVQPGGSLRLSCAAT | MGWYRQAPGKQRELVAS | T | NYADSVKGRFTISRENAKNQVYLQMNSLQPEDTAVYYC | WGQGTQVTVSS | 9.7 |
| VHH43 | - | QVQLVESGGGLVQAGGSLRLSCAAS | MAWFRQAPGKEREFLAV | T | YYANSVKGRFTISRDDAKNTLFLQMNSLKPEDTAVYYC | WGQGTQVTVSS | 18.2 |
| VHH44 | - | QVKLEESGGGLVQAGDSLRVSCAAS | MGWFRQAPGKEREFVAA | T | RYADSVKGRFTISRDNDKNMVYLQMNSLKPEDTAVYYC | WGQGTQVTVSS | 33 |
| ICAM11-4 | - | QVQLVESGGGLVQPGGSLRLSCAAS | MGWYRQAPGKQRELVAD | I | YYVDSLKGRFTISRDNARSTVYLQMNSLEPEDTAVYYC | WGQGTQVTVSS | 1.1 |
| ICAM34-1 | - | QVKLEESGGGLVQPGGSLRLSCAAS | MGWYRQAPGKQRELVAR | A | AYEDSVKGRFTISRDNAPNTVFLQMNGLKPEDTAVYYC | WGQGTQVTVSS | 2.2 |
| IGF1R-4 | - | QVKLEESGGGLVQAGGSLRLSCEVS | MGWFRQAPGKEREFVGH | T | RVASSVKDRFTISRDSAKNTVYLQMNSLKSEDTAVYYC | WGQGTQVTVSS | 0.3 |
| IGF1R-5 | - | QVKLEESGGGLVQAGGSLRLSCAAS | MAWSRQAPGKDREFVAT | A | RYANSVKGRFTISRDNAKGTMYLQMNNLEPEDTAVYSC | WGQGTQVTVSS | 0.2 |
| VHH49 | - | QVKLEESGGGLVQPGGSLRLSCAAS | MTWVRQSPGKGLEWVSA | T | SYSDSVKGRFTISRDNAKNTLYLQMNSLKPEDTAMYYC | WGQGTQVTVSS | 20.3 |
| VHH50 | - | QVKLEESGGGLVQPGGSLRISCAAS | MSWVRQAPGKGLEWVST | A | YYADSVKGRFTISRDNARNTLYLQMNSLKPEDTAVYYC | WGQGTQVTVSS | 1.8 |
| VHH51 | - | QVQLVESGGGLVQAGGSLRLSCAKS | AGWYRQAPGKQRELVAT | T | NYADSVKGRFTISRDSAKNAVYLQMNSLKPEDTATYYC | WGQGTQVTVSS | 111 |
| VHH52 | - | QVKLEESGGGLVQAGGSLRLSCKAS | MGWFRQAPGKEQEFVAA | T | KYADAVKGRFTISRDNPKNTENTVSLQMNSLKPEDTAVYHC | RGQGTQVTVSS | 46 |
| VHH53 | - | QVKLEESGGGLVQAGGSLRVSCTAS | LGWYRQAPGKARELVAA | V | YYTDSVKGRFTISRDNAKNTVSLQMSSLKPEDTAVYYC | WGQGTQVTVSS | 1.3 |
| VHH54 | - | QVKLEESGGGLVQAGGSLRLSCAAS | MGWFRQAPGEEREFVAA | K | TYADSVKGRFTISRDNPKNTMYLQMNSLKPEDTAVYYC | WGQGTQVTVSS | 35.2 |
| VHH55 | CDR1-CDR3 | DVQLVESGGGSVQAGGSLRLSCAVS | TGWYRQAPGKEREWVSS | I | YYQDSVKGRFTISRDNAKNTVYLQMNSLQREDTGMYYC | WGQGTQVTVSS | 0.5 |

SpA contact residues are highlighted in yellow. Amino acid substitutions at SpA contact positions are shown in red.

^*^Values are from 500 nM injections (all others were from 250 nM injections). The values from 500 nM injections were divided by 2 for plotting in main text Fig. 2.
