# Supplementary material for: A Rational Engineering Strategy for Designing Protein A-Binding Camelid Single-Domain Antibodies
Source: PLoS One. 2016 Sep 15;11(9):e0163113. doi: 10.1371/journal.pone.0163113 (PMC5025174; doi:10.1371/journal.pone.0163113)
Supplement: S2 Table — (DOCX) [file pone.0163113.s006.docx]

**S2 Table**. FR sequences of SpA-binding and non-SpA-binding V_H_H pentamers.

| **V_H_H** | **Disulfide Bonds** | **FR1** | **FR2** | **CDR2** | **FR3** | **FR4** | **SpA Binding (RU bound at end of injection)** |
| --- | --- | --- | --- | --- | --- | --- | --- |
| Pentamer1 | - | QVKLEESGGGLVQVGGSLRLSCTAS | MGWFRQPPGKEREIVAA | T | LYGESVKGRFTVSRDNAKNTVYLQMNSLKPEDTAVYYC | WGQGTQVTVSS | 2270 |
| Pentamer2 | - | QVQLVESGGGLVQPGGSLRLSCVAS | IAWFRQAPGKGLEWLSV | T | NYADSVKGRFTISRDNAKNTVYLQMNSLKPEDTAVYSC | WGQGTQVTVSS | 866 |
| Pentamer3 | - | QVKLEESGGGLVQAGGSLSLSCAAT | MGWFRQAPGKERDFIAA | T | YYADSVKGRFTISRDNAKNEVYLQMNSLKPEDTAVYYC | WGQGTQVTVSS | 846 |
| Pentamer4 | FR2-CDR3 | EVQLQASGGGLVQPGGSLRLSCAAS | IGWFRQAPGKEREGVSC | T | YYADSVKGRFTISRHNAK-TVYLQMNSLKPEDTAVYYC | WGQGTQVTVSS | 634 |
| Pentamer5 | - | QVKLEESGGGLVPPGGSLRLSCEAS | MYWYRQAPGKQRELVAS | T | TYSDSVKGRFTISGDRVKNEVYLQMNSLKPEDTAVYYC | WGQGTQVTVSS | 619 |
| Pentamer6 | - | QVKLEESGGGSVQAGGSLRLSCAAS | MAWFRQAPGKEREGVGK | T | YYADSVKGRFAISRDNSNDRAYLQMNNLRPEDTGVYYC | WGQGTQVTVSS | 585 |
| Pentamer7 | - | QVKLEESGGGSVQAGGSLRLSCAAS | MAWFRQAPGKEREGVGK | T | YYADSVKGRFTISRDNSNDRAYLQMNNLRPEDTGVYYC | WGQGTQVTVSS | 583 |
| Pentamer8 | - | QVKLEESGGGLVQAGGSLRLSCAAS | MAWYRRLPGKHRDLVAV | T | NYADSVKGRFTISRDITKNTVDLQMSNLKPEDTAVYYC | WGQGTQVTVSS | 534 |
| Pentamer9 | - | DVQLQASGGGLVQAGGSLRLSCAAS | IGWYRVPPGKQRESVAA | A | NYLDSVKGRFTISRDNAKNTVYLQMNSLKPEDTAVYYC | LGQGTQVTVSS | 439 |
| Pentamer10 | FR2-CDR3 | QVKLEESGGGLVQAGGSLRLSCRVS | IGWFRLAPGKDREGVAC | T | YYADSVKGRFTISRDNAKNAVYLQMSSLKPDDTAVYYC | WGQGTQVTVSS | 399 |
| Pentamer11 | - | QVKLEESGGGLVQAGGSLRLACAAS | MGWFRQAPGKEREFVSR | T | SYADSVKGRFSISRDNAKNMVFLQMNSLKPEDTAVYYC | WGQGTQVTVSS | 351 |
| Pentamer12 | - | QVQLVESGGGSVQAGGSLRLSCAAS | LGWFRQAPGKEREGVAS | S | YYADSVRGRFTISRDNDKNTVYLQMNSLKPEDTAIYYC | WGQGTQVTVSS | 340 |
| Pentamer13 | - | QVKLEESGGGLVQAGGSLRLSCAAS | MGWFRQAPGTEREFVAT | T | VYASSVKGRFTISRDDAKSTVYLQMNSLKPEDTAVYYC | WGQGTQVTVSS | 331 |
| Pentamer14 | - | QVKLEESGGGLVQAGGSLRLSCAAS | MYWFRQAPGKEREFVAA | T | YYTDSVKGRFTISRDNAKNTVYLQMNSLKPEDTAVYYC | WGQGTQVTVSS | 233 |
| Pentamer15 | FR2-CDR3 | QVKLEESGGGLVQPGGSLTLSCTLS | VGWFRQAPGKEREGVSC | T | YYRESVKGRFTISRDNAKNSVTLRMNNLRPEDTAVYYC | WGQGTQVTVSS | 205 |
| Pentamer16 | - | QVQLVESGGGLVHPGGSLRLSCATS | MRW-RQAPGKGLEWVSD | T | TYANFAKGRFTISRDNAERTVYLYMNSLAPVDTAVYYC | WGQGTQVTVSS | 174 |
| Pentamer17 | FR2-CDR3 | EVQLQASGGGLVQPGGSLRLSCAAS | IGWFRQAPGKAREGVAC | A | DYTDSVKGRFTISRDIARNTVYLQMNSLKPEDTAVYYC | WGQGTQVTVSS | 57 |
| Pentamer18 | - | QVKLEESGGDLVQPGGSLRLSCAAS | MGWFRQAPGKQRELVAT | T | NYAYSVKDRFTVSRDNAKNTLYLQMNDLKPEDTAVYYC | WGQGTQVTVSS | 118 |
| Pentamer19 | - | DVQLQASGG-VVQPGGSLRLSCAAH | MGWGRQAPGKQREYVAT | T | NYASSVEGRFTISRDNAKKTVYLQMNDLKPEDTAVYYC | WGQGTQVTVSS | 5 |

SpA contact residues are highlighted in yellow. Amino acid substitutions at SpA contact positions are shown in red.
