# Supplementary material for: A Rational Engineering Strategy for Designing Protein A-Binding Camelid Single-Domain Antibodies
Source: PLoS One. 2016 Sep 15;11(9):e0163113. doi: 10.1371/journal.pone.0163113 (PMC5025174; doi:10.1371/journal.pone.0163113)
Supplement: S3 Table — (DOCX) [file pone.0163113.s007.docx]

**S3 Table.** FR sequences of five non-SpA-binding V_H_Hs and their humanized SpA-binding counterparts.

| V_H_H | **FR1** | **FR2** | **CDR2** | **FR3** | **FR4** |
| --- | --- | --- | --- | --- | --- |
| **HVH430** | QVQLVESGGGLIKPGGSLRLSCAAS | MSWVRQAPGKGLEWVSA | T | YYADSVKGRFTISRDNSKNTVYLQMNSLRAEDTAVYYC | WGQGTMVTVSS |
| **IGF1R-4 (llama, - SpA)** | QVKLEESGGGLVQAGGSLRLSCEVS | MGWFRQAPGKEREFVGH | T | RVASSVKDRFTISRDSAKNTVYLQMNSLKSEDTAVYYC | WGQGTQVTVSS |
| **IGF1R-4 (humanized, + SpA)** | QVQLVESGGGLVQPGGSLRLSCAVS | MGWFRQAPGKGLEFVGH | T | RYASSVKGRFTISRDNSKNTVYLQMNSLRAEDTAVYYC | WGQGTLVTVSS |
| **IGF1R-5 (llama, - SpA)** | QVKLEESGGGLVQAGGSLRLSCAAS | MAWSRQAPGKDREFVAT | A | RYANSVKGRFTISRDNAKGTMYLQMNNLEPEDTAVYSC | WGQGTQVTVSS |
| **IGF1R-5, (humanized, + SpA)** | QVQLVESGGGLVQPGGSLRLSCAAS | MAWSRQAPGKGLEFVAT | T | RYANSVKGRFTISRDNSKNTMYLQMNSLRAEDTAVYYC | WGQGTLVTVSS |
| **ICAM11-4 (llama, - SpA)** | QVQLVESGGGLVQPGGSLRLSCAAS | MGWYRQAPGKQRELVAD | I | YYVDSLKGRFTISRDNARSTVYLQMNSLEPEDTAVYYC | WGQGTQVTVSS |
| **ICAM11-4 (humanized, + SpA)** | QVQLVESGGGLVQPGGSLRLSCAAS | MGWYRQAPGKGLELVAD | T | YYVDSLKGRFTISRDNSKNTVYLQMNSLRAEDTAVYYC | WGQGTLVTVSS |
| **ICAM34-1 (llama, - SpA)** | QVKLEESGGGLVQPGGSLRLSCAAS | MGWYRQAPGKQRELVAR | A | AYEDSVKGRFTISRDNAPNTVFLQMNGLKPEDTAVYYC | WGQGTQVTVSS |
| **ICAM34-1 (humanized, + SpA)** | QVQLVESGGGLVQPGGSLRLSCAAS | MGWYRQAPGKGLELVAR | T | AYEDSVKGRFTISRDNSKNTVYLQMNSLRAEDTAVYYC | WGQGTLVTVSS |
| **AFAI (- SpA)** | DVQLQASGG-VVQPGGSLRLSCAAH | MGWGRQAPGKQREYVAT | T | NYASSVEGRFTISRDNAKKTVYLQMNDLKPEDTAVYYC | WGQGTQVTVSS |
| **AFAI (+ SpA)** | DVQLQASGG-VVQPGGSLRLSCAAH | MGWGRQAPGKQREYVAT | T | NYASSVKGRFTISRDNAKKTVYLQMNSLKPEDTAVYYC | WGQGTQVTVSS |

SpA contact residues are highlighted in yellow. Amino acid substitutions at SpA contact positions introduced in humanized V_H_Hs are shown in red, and substitutions outside SpA contact positions are shown in green. An unusual Pro residue at position 75 of V_H_H ICAM34-1, and its substitution by Lys75 in the humanized V_H_H, are highlighted in pink.
