# Supplementary material for: A Rational Engineering Strategy for Designing Protein A-Binding Camelid Single-Domain Antibodies
Source: PLoS One. 2016 Sep 15;11(9):e0163113. doi: 10.1371/journal.pone.0163113 (PMC5025174; doi:10.1371/journal.pone.0163113)
Supplement: S4 Table — (DOCX) [file pone.0163113.s008.docx]

**S4 Table.** Metrics for Illumina MiSeq NGS data used in this study.

|  | **Llama 1** | **Llama2** | **Llama3** | **Alpaca, camel, llama** |
| --- | --- | --- | --- | --- |
| No. of raw reads | 101,219 | 659,761 | 1,473,175 | 2,155,690 |
| No. of assembled reads (FLASH) | 61,823 | 273,953 | 929,131 | 915,274 |
| No. of reads passing quality filter (FASTX) | 45,464 | 200,031 | 647,192 | 652,932 |
| No. of reads analyzed | 43,038 | 164,686 | 604,026 | 451,010 |
