# Supplementary material for: A Rational Engineering Strategy for Designing Protein A-Binding Camelid Single-Domain Antibodies
Source: PLoS One. 2016 Sep 15;11(9):e0163113. doi: 10.1371/journal.pone.0163113 (PMC5025174; doi:10.1371/journal.pone.0163113)
Supplement: S5 Table — (DOCX) [file pone.0163113.s009.docx]

**S5 Table**. FR and CDR sequences of a SpA-binding (Thr57) and non-SpA-binding (Ile57) dromedary V_H_H and effect of CDR1-CDR3 disulfide bridge on SpA binding.

| **V_H_H** | **Disulfide Bonds** | **FR1** | **CDR1** | **FR2** | **CDR2** | **FR3** | **CDR3** | **FR4** | **SpA Binding (RU bound at end of injection)** |
| --- | --- | --- | --- | --- | --- | --- | --- | --- | --- |
| VHH55 | CDR1-CDR3 | DVQLVESGGGSVQAGGSLRLSCAVS | GSTYSPCT | TGWYRQAPGKEREWVSS | ISSPGTI | YYQDSVKGRFTISRDNAKNTVYLQMNSLQREDTGMYYC | QIQCGVRSIREY | WGQGTQVTVSS | 0.5 |
| VHH55.1 | CDR1-CDR3 | DVQLVESGGGSVQAGGSLRLSCAVS | GSTYSPCT | TGWYRQAPGKEREWVSS | ISSPGTT | YYQDSVKGRFTISRDNAKNTVYLQMNSLQREDTGMYYC | QIQCGVRSIREY | WGQGTQVTVSS | 69.4 |
| VHH55.2 | - | DVQLVESGGGSVQAGGSLRLSCAVS | GSTYSPAT | TGWYRQAPGKEREWVSS | ISSPGTT | YYQDSVKGRFTISRDNAKNTVYLQMNSLQREDTGMYYC | QIQAGVRSIREY | WGQGTQVTVSS | 60.8 |
| VHH55.3 | - | DVQLVESGGGSVQAGGSLRLSCAVS | GSTYSPAT | TGWYRQAPGKEREWVSS | ISSPGTI | YYQDSVKGRFTISRDNAKNTVYLQMNSLQREDTGMYYC | QIQAGVRSIREY | WGQGTQVTVSS | 0.8 |
